# Experiences of women living with HIV who underwent long loop excision of the transformation zone (LLETZ) in selected hospitals of Vhembe district, Limpopo province, South Africa

**DOI:** 10.12688/f1000research.158796.2

**Published:** 2025-09-05

**Authors:** Mabareki Cecilia Machaba Sikhwetha, Lufuno Makhado

**Affiliations:** 1 Public Health, Faculty of Health Sciences, University of Venda, Thohoyandou, Limpopo, 0950, South Africa

**Keywords:** Experiences, Human Immunodeficiency Virus, Large Loop Electrosurgical removal of the Transformation Zone, premalignant, Reproductive age.

## Abstract

**Background:**

Cervical cancer poses a significant health threat, especially for women living with HIV, who are at increased risk of developing premalignant cervical lesions. Large Loop Excision of the Transformation Zone (LLETZ) is a standard treatment for cervical intraepithelial neoplasia (CIN) to prevent progression to malignancy. This study explores the experiences of HIV-positive women who underwent LLETZ in Vhembe District, South Africa, where access to such specialized treatments remains limited.

**Methods:**

Using a qualitative, phenomenological approach, this study engaged seven HIV-positive women who underwent LLETZ, capturing their lived experiences through in-depth, unstructured interviews. Thematic analysis identified key themes and subthemes to elucidate their psychological, physical, and social encounters related to the procedure.

**Results:**

Analysis revealed eight central themes: psychological and physical experiences, psychosocial and financial support, misconceptions, education, recommendations, and time-related factors. Participants reported anxiety, fear, and initial stress about the procedure, coupled with resilience and acceptance over time. Physical effects such as pain and bleeding were common, though recovery experiences varied. Social support from family and community played a critical role in coping, while financial constraints impacted access to and continuity of care. Misunderstandings about the LLETZ procedure underscored the need for enhanced patient education. Participants emphasized timely screening and support systems to improve treatment outcomes and reduce psychological distress.

**Conclusions:**

The study highlights the multifaceted impact of LLETZ on HIV-positive women, including the psychological and financial burdens and the importance of clear communication and patient education. Improved support structures, timely result communication, and access to follow-up care are essential to enhance patient outcomes. Recommendations advocate for health system improvements, partner involvement, and proactive counselling to address the specific needs of HIV-positive women undergoing cervical cancer prevention treatments.

## Introduction

Globally, cervical cancer is the fourth most common cancer among women, with an estimated 311,365 deaths reported annually ( Zou et al., 2020). HIV-positive women are particularly susceptible to cervical intraepithelial neoplasia (CIN), necessitating timely and effective interventions like LLETZ. Despite its efficacy, limited research captures women's lived experiences undergoing this procedure. Understanding these experiences is essential to contextualize the role of LLETZ in high-risk populations and to identify gaps in current clinical recommendations.

Large Loop Excision of the Transformation Zone (LLETZ) is the preferred treatment for cervical intraepithelial neoplasia due to its effectiveness and ability to be performed in an outpatient setting under local anaesthesia (LA) ( Yap et al., 2020). In HIV-negative women, cure rates exceed 90%. However, outcomes are poorer in HIV-positive cohorts, where pooled treatment failure rates reach 21.4%, corresponding to a success rate of 78.6% Debeaudrap et al. (2019). This discrepancy highlights the importance of examining not only clinical outcomes but also the personal experiences of women who undergo the procedure. The goal of 2030 is to reduce premature mortality from non-communicable diseases, including cervical cancer, by one-third ( WHO, 2014). Achieving this goal is both feasible and sustainable.

In the sub-Saharan region ( Chambuso et al., 2017) have reported a highly statistically significant association (P < .001) between HIV/AIDS and the development of cervical precancerous lesions in HIV-positive women and advocate for LLETZ as one of the main approaches for treating cervical intraepithelial neoplasia grade 2–3 (CIN2+) precancerous lesions. Debeaudrap et al. (2019) present strong evidence for an increased risk of treatment failure in HIV-infected women compared to their HIV-negative counterparts. However, among 40 eligible studies, the pooled prevalence of treatment failure in HIV-infected women was 21.4%, suggesting a considerable treatment success rate of 78.6%. Despite this, the absence of tailored recommendations for follow-up care, recurrence management, and long-term monitoring in HIV-positive women underscores the need for scientific evidence to guide optimal interventions. Any surgical procedure has its effect/s on the one undergoing it, be it desired or adverse. LLETZ is not exceptional.

Cervical cancer is an identified national priority in South Africa and other sub-Saharan countries and the second most common cancer among women in South Africa after breast cancer. It ranks as the leading cause of female cancer deaths in South Africa, particularly affecting women in the reproductive age group between 15 and 44 years ( Marañón et al., 2017). There is a notable absence of recorded experiences from women, both those living with HIV and those without, following LLETZ procedures. The National Cervical Cancer Policy mandates annual pap smears for HIV-positive women in Vhembe District to screen for premalignant lesions ( Marañón et al., 2017). Primary healthcare facilities administer these screenings, and those who show abnormal results are referred to hospitals for further management. However, the challenge lies in the limited availability of hospitals offering LLETZ services, with only two selected for this study. Furthermore, the scarcity of specialized nurses for LLETZ procedures adds to the complexities of delivering these critical services. The study took place in Vhembe District, Limpopo Province, South Africa, in two rural public hospital settings offering LLETZ services to needy women. The two hospitals were the only public ones providing the service in the district, constituting a population of approximately 1402,779. Of this population, 54% are women, and 51% of households are women-headed, slightly exceeding the rates in Limpopo (48.85%) and significantly higher than the national average in South Africa (41.32%) ( Vhembe District Municipality, 2020). Thus, addressing the quality of life for these women becomes crucial. This emphasis is essential to enhance their life expectancy, enabling them to perform their responsibilities as mothers effectively. Notably, the district has a high uninsured population of 93.6%, reliant on the public health sector for their healthcare needs ( Vhembe District Municipality, 2020). Taken together, these challenges illustrate that beyond clinical data, there is limited knowledge about how HIV-positive women experience and cope with LLETZ treatment, especially in rural and resource-limited healthcare settings.

There are gaps in our understanding of this phenomenon, e.g. although there is evidence indicating that HIV-positive women of reproductive age are often diagnosed with premalignant lesions and undergo LLETZ treatment, specific recommendations for their care are lacking. Global research has highlighted some expressed and observed experiences, advocating for a dedicated treatment protocol for this group. Despite facing these challenges, they are often heads of families.

The purpose of this qualitative phenomenological study was to explore and describe the experiences of women of reproductive age living with HIV who had undergone LLETZ within the district by capturing their voices through the employment of in-depth, unstructured interviews. The outcome has the potential to fill the identified gap in the literature, adding to the understanding of the phenomenon and providing more insight, thus improving practice as unheard voices are considered. The study was conducted in the year 2024. Women living with HIV, compared to those living without HIV, are considered a high-risk group for the development of pre-malignant lesions. Furthermore, LLETZ-related services are scarce in Vhembe, necessitating affected women to wait on a list, during which time CIN2-3 may progress into malignancy. Due to the scientific evidence that women lead more than 50% of households, this can contribute to a generally burdened and unhealthy society. Thus, to achieve the goal of health for all by 2030, it is essential to promote women’s health by, amongst others, mitigating such potential challenges. Against this background, the purpose of this qualitative phenomenological study was to explore and describe the experiences of women of reproductive age living with HIV who had undergone LLETZ within the district. Through capturing their voices through in-depth, unstructured interviews, the study aims to fill this gap in the literature and generate insights to inform more patient-centred care.

## Methods

### Study design

This qualitative study employed a phenomenological approach (PA) as its conceptual framework to explore the personal experiences of women living with Human Immunodeficiency Virus (HIV) who have undergone Large Loop Excision of the Transformation Zone (LLETZ). The phenomenological approach is particularly effective in capturing the nuances of these women's experiences, as it seeks to understand the essence of their situations by asking, “What is this or that kind of experience like?” Unlike other qualitative methods, phenomenology aims for a descriptive understanding of pre-reflective experiences without imposing categories, classifications, explanations, or meanings ( Van Manen, 2017).

The study specifically focused on women of reproductive age living with HIV, going beyond typical qualitative inquiries to provide a rich, detailed account of their experiences with LLETZ. The roots of phenomenology trace back to the early 20th century, with significant contributions from philosophers such as Heidegger and Gadamer, while also being grounded in the foundational work of Edmund Husserl ( Neubauer et al., 2019). This framework asserts that "lived experiences are an interpretative process situated in an individual's lifeworld," where the observer inherently influences understanding through their unique perspective ( Neubauer et al., 2019). Additionally, Bynum and Varpio (2018) highlight that phenomenology seeks to uncover deeper layers of human experience, which often remain hidden beneath conscious awareness. This study also explored how an individual's lifeworld—defined as the world as it is pre-reflectively experienced—affects these obscured dimensions. The researchers actively engaged in the interpretive process, as Tuffour (2017) emphasised, providing an essential aspect of phenomenological research.

The study design was exploratory-descriptive, focusing specifically on LLETZ interventions. In-depth, one-on-one, unstructured interviews were conducted to collect comprehensive and insightful accounts of the women's experiences. These interviews yielded rich descriptions that illuminate the realities faced by this population, aligning with the standards set by the SRQR (Standards for Reporting Qualitative Research) guidelines, which emphasize the importance of transparency, rigor, and the context of qualitative research.

### Research paradigm

This study was conducted within an interpretive framework, which acknowledges the inherently subjective nature of individual experiences. It emphasizes that understanding these experiences is influenced by the perspectives and interpretations of the researchers involved in the study. By recognizing the researchers' role in the interpretative process, the study aims to provide a deeper insight into the complexities of the participants' experiences rather than merely presenting objective facts. This approach highlights the importance of context and personal interpretation in understanding the explored phenomena.

### Study setting

The study was conducted in Vhembe District, Limpopo Province, South Africa. Vhembe is situated in the Northern part of Limpopo Province, sharing borders with Capricorn and Mopani districts in the eastern and western directions, Mozambique through the Kruger National Park in the south-east, Zimbabwe in the north and Botswana in the northwest. The district comprises four local municipalities: Thulamela, Makhado, Musina and the recently established Collins Chabane local municipality. Of 387 089 households, 51% are female-headed, totalling 194,980 ( Vhembe District Municipality, 2020). Of 149 healthcare facilities within the district, two selected hospitals offer LLETZ services to women with premalignant lesions. All facilities refer women to these two hospitals for LLETZ. This study was conducted in private rooms in outpatient departments of the selected hospitals.

### Study population, sampling and recruitment

The study focused on a specific group of women living with Human Immunodeficiency Virus (HIV) who underwent LLETZ in selected hospitals within the Vhembe District of Limpopo Province, South Africa.

***Sampling technique***

This study employed a purposive sampling technique, allowing researchers to select participants based on specific characteristics relevant to the phenomenon under study. According to Brink et al. (2014), purposive sampling is grounded in the researcher's judgment to identify individuals who are either typical representatives of the study phenomenon or possess specialized knowledge. In this study, the selected participants were women aged 18 to 44 years, living with HIV, who had previously undergone the Large Loop Excision of the Transformation Zone (LLETZ) procedure.

***Recruitment process***

The recruitment process occurred in selected hospitals' outpatient departments (OPD) during gynaecology clinic days. To identify potential participants, the researcher collaborated with the nurse assisting the gynaecologist, who was requested to refer eligible women to the researcher. This ensured that only those meeting the inclusion criteria were approached. Once referred, the researcher invited each potential participant to a private consultation room to ensure confidentiality and comfort. The researcher introduced herself, explained the purpose of the study, and provided details about the in-depth, unstructured interviews. Participants were given ample time to ask questions, and informed consent was obtained before proceeding. To initiate the interview, participants were first asked about their understanding of the LLETZ procedure, followed by the central research question.

***Sample size determination***

The sample size was determined by data saturation, the point at which no new information emerged from additional interviews. Although the initial plan was to interview seven participants, including two for a pretest, the study was concluded when saturation was reached with the seventh participant. The two pretest participants were included in the final analysis, as their contributions were relevant and aligned with the study's objectives.

***Ethical considerations***

This study adhered to strict ethical guidelines to ensure participants' protection, rights, and well-being. The research received approval through a rigorous multi-step process. It was first reviewed by the Department of Public Health at the University of Venda, followed by the Faculty of Health Sciences Higher Degrees Committee and the University Higher Degrees Committee, before final approval from the University of Venda Human and Clinical Trial Research Ethics Committee, which granted an Ethical Clearance Certificate (Ethical Clearance no: **FHS/23/PH/56/1801**). Further permission was sought through the National Health Research Database (NHRD) from the Limpopo Department of Health (DoH) to conduct the study in two selected hospitals in Vhembe District (Ref: **LP_2024-02-013**). Approval was granted on the condition that a formal permission letter be submitted to the Vhembe District Health Department one week before data collection. The district health department subsequently issued a permission letter, which was presented to the hospital management. Before data collection, the researcher reported to the Operational Manager’s office in the OPD to ensure transparency.

Written Informed consent was obtained after participants were thoroughly informed about the study's purpose, procedures, and expectations. They were assured of their right to withdraw at any time without consequences, and confidentiality was emphasized, ensuring that their identities would not be disclosed. Participants voluntarily signed consent forms, confirming their willingness to participate. The principle of beneficence was upheld by ensuring emotional well-being through support from social workers and psychologists in the selected hospitals. The researcher remained observant for signs of distress during interviews and allowed participants to express concerns. Vulnerable individuals or those experiencing acute distress were excluded to prevent harm. Confidentiality was maintained through data coding (e.g., P1, P2, P3) to uphold the principle of justice, preventing linkage between responses and participants' identities. Names appeared only on consent forms, which were securely stored and discarded after the study. The consent form was also translated into Tshivenda, Xitsonga, and Sepedi to accommodate non-English-speaking participants.

### Data collection tool and method

***In-Depth unstructured interviews***

This study employed a self-report technique, utilizing in-depth unstructured interviews to explore participants' experiences with LLETZ. Each interview took place in a private room within the selected hospitals, ensuring confidentiality. The researcher encouraged open-ended discussions, using probing and prompting techniques to allow participants to elaborate on their experiences. A central question guided the interviews:

*"What is it like to be an HIV-positive woman who has undergone LLETZ, a surgical procedure where a tissue sample is taken from the cervix to remove identified premalignant cells and tested to exclude cancer?"*

To maintain consistency, the researcher followed a structured approach, using paraphrasing and clarification techniques to align the interaction across all participants. Audio recordings supported data collection to capture the conversations accurately. Additionally, field notes documented non-verbal cues, enhancing the depth of data interpretation. Bracketing techniques ensured the researcher remained objective, reducing potential biases and capturing an authentic account of participants’ experiences ( Alase, 2017). The study utilized semi-structured interview guides in the supplementary materials included with this paper for further review (See supplement 1).

***Pretest***

A pretest was conducted to evaluate the clarity and effectiveness of the interview questions and data collection tools. Two participants from the target population participated in this phase, and their responses were included in the final study as their insights were deemed relevant and valuable. The pretest phase also allowed refinement of probing techniques, ensuring that the interview questions elicited detailed narratives rather than simple ‘yes’ or ‘no’ responses.

### Measures to ensure trustworthiness

***Credibility***

Credibility was ensured through rigorous methodological adherence, with ethical considerations guiding all stages of the research. Institutional relationships were established before interviews commenced, ensuring smooth access to participants. Data collection combined in-depth interviews with observational field notes, allowing for a comprehensive exploration of LLETZ experiences. A purposive sampling technique was applied to ensure that participants were appropriately selected. The study adhered to the inclusion and exclusion criteria outlined in Section 3.4.3. Data robustness was reinforced through cross-checking findings with existing literature, ensuring alignment with broader research insights. Data saturation was confirmed as no new themes emerged by the seventh interview.

***Transferability***

The study parameters were clearly defined to support transferability, allowing future researchers to determine applicability to other contexts. The study was conducted in two purposefully selected hospitals in Vhembe District, Limpopo Province, as they were the only facilities offering LLETZ services in the region. The research timeline covered one month in 2024, with seven interview sessions lasting 30 to 50 minutes. Though the findings may not be universally transferrable, they provide a valuable foundation for comparison in future related studies.

***Dependability***

The research process was documented to ensure dependability, including detailed accounts of the research design, data collection, and analysis ( Brink et al., 2014). Primary data sources, including audio recordings (AR), field notes (FN), transcripts (T), and reflexivity notes (RN), were archived systematically for transparency and traceability of the research process. A predefined data management and analysis plan guided the data collection. The rigorous documentation ensured consistency, allowing future studies to replicate or validate findings.

***Confirmability***

The researcher ensured objectivity and neutrality by setting aside personal interpretations during data collection. Subjective reflections were only revisited during data analysis and interpretation, preventing undue influence on participants’ responses. Field notes captured non-verbal expressions cross-referenced with audio transcripts and reflexivity notes to ensure a comprehensive dataset. Triangulation was applied through multiple data sources, and findings were validated against established literature, strengthening the study’s credibility and reliability. The researcher’s background as an oncology nurse specialist in colposcopy clinics, alongside supervisory guidance, contributed to the study’s credibility and expertise-driven approach.

### Data collection

Data were collected in outpatient departments of the selected hospitals in Vhembe District, Limpopo Province. To facilitate smooth research operations, permission letters were submitted directly to the Registry Departments of the selected hospitals rather than the Chief Executive Officers (CEOs), as per the institutions' administrative structures. Requests were also made for a private interview room and approval to engage hospital psychologists and social workers in case of participant distress. On gynaecology clinic days, the researcher worked with nurses assisting the gynaecologist in identifying eligible participants based on the study’s inclusion criteria. Once identified, women were referred to a private interview room, where in-depth, unstructured interviews were conducted.

Each interview followed a structured approach, beginning with welcoming and greeting the participant, researcher self-introduction, study purpose explanation, and consent acquisition. Participants were asked to share their understanding of LLETZ to initiate discussion, followed by the central research question. Interviews were conducted in a language preferred by the participant, ensuring clarity and comfort. Probing, paraphrasing, and follow-up questions were applied based on participants’ responses and non-verbal cues. Each interview concluded with the researcher confirming the accuracy of the participant’s responses, reiterating confidentiality, and expressing gratitude for their contributions. All interviews were conducted on the same day to minimize logistical challenges and travel expenses. Each session lasted 30 to 50 minutes, and data saturation was confirmed after seven interviews. One additional participant was interviewed instead of the originally planned two to accommodate recruitment challenges, ensuring no new insights emerged beyond the seventh interview.

To validate responses, member checking was conducted throughout the interviews. Field notes were taken in real-time, and audio recordings were transcribed and documented in reflexive journals after each session, away from participants ( Alase, 2017). The study specifically examined women living with HIV and their experiences with LLETZ procedures in 2024, ensuring that findings reflect recent clinical and social realities within the targeted demographic.

### Data organization and preparation for analysis

The organization and preparation of data for analysis were done as follows: Items of information gathered included audio records (AR) and field notes (FN), which provided significant reflections of non-verbal cues demonstrated by each participant (P). Transcripts (T) were created from translated voice recordings. Reflexive notes (RN) of each participant represent the researcher`s reflections on collected data done away from the interview site. These were organized/grouped without using the real names of participants. Instead, they were arranged in the sequence of interviews, labeled as P1, P2, P3 etc. The first group, P1, consisted of (AR1, FN1, T1 and RN1), corresponding to the participant interviewed first. The data for subsequent participants followed the same pattern, with P2 (AR2, FN2, T2 and RN2) for the second participant and so on. This organized data will be kept in the University Library. The Tesch coding procedure was implemented in the thematic analysis ( Creswell & Creswell, 2017).

### Data analysis

The data collected in this study was analyzed using Tesch’s thematic coding procedure, a structured approach that ensures a comprehensive and rigorous thematic analysis ( Creswell & Creswell, 2017, 2018). This method involved systematic transcription, coding, categorization, and theme development, allowing in-depth exploration of participants' lived experiences with LLETZ.

The analysis began with the repeated listening and transcription of audio recordings from each participant. The transcriptions were completed in English, and each participant's written text file (T) was generated in Microsoft Word. Each transcript was then carefully examined alongside field notes (FN), which captured non-verbal expressions, behavioral observations, and contextual details recorded during interviews. Additionally, reflexive notes (RN) were written for each participant on the day of the interview, away from the participant, to document the researcher’s reflections, interpretations, and observations. To extract meaningful insights, all transcripts, field notes, and reflexive notes were read and re-read multiple times to identify key patterns, emerging ideas, and recurring concepts. A color-coding technique was applied to organize these patterns into initial themes, ensuring that each emerging topic was systematically categorized (Annexure IX). The iterative reading process further refined the analysis, allowing for a deeper understanding of how each category interrelated and contributed to the overall dataset. This process also facilitated the identification of sub-themes, ensuring that nuanced aspects of participants’ experiences were adequately captured.

Once the primary themes and sub-themes were established, all coded data material corresponding to each theme was gathered, reviewed, and analyzed in-depth. Throughout this process, the researcher maintained a critical awareness of personal preconceptions and biases, employing bracketing techniques to ensure that the findings authentically reflected participants' experiences rather than researcher assumptions. A final re-reading of the entire dataset was conducted to verify that all relevant data had been appropriately classified. No unclassified items remained, confirming the completeness and consistency of the thematic analysis. This structured and rigorous analytical process ensured that the study's findings were credible, transferable, and deeply rooted in participants’ lived realities.

## Results

During data analysis, no new data emerged after Participant Seven, indicating that saturation was reached. This means that the experiences of women living with HIV who underwent LLETZ in selected hospitals of Vhembe District, Limpopo Province, South Africa, were adequately represented.

***Demographic data***

This study comprised seven women of reproductive age who attended the gynaecology clinic in OPDs in hospitals in the Vhembe District. All participants were living with HIV, had pap smears resulting in premalignant lesions and underwent LLETZ as an intervention treatment.

***Thematic analysis of experiences of women living with HIV undergoing LLETZ treatment***

Thematic analysis of the transcripts from women living with HIV who underwent LLETZ treatment revealed eight primary themes ( Table 1): LLETZ Intervention Process and Outcomes, Psychological Experiences, Physical Experiences, Psychosocial Support, Financial Support, Misconceptions and Education, Recommendations and Reflections and Time Factor. Four of the themes included several sub-themes, which captured the nuanced experiences of the participants.

**Table 1.** Themes and sub-themes.

Theme	Sub-Theme
LLETZ Intervention Process and Outcomes	
Psychological Experiences	Acceptance and Resilience
	Anxiety and Stress
	Uncertainty about Health Status and Future
Physical Experiences	Pain and Discomfort
	Recovery Process
Psychosocial Support	Family and Partner Support
	Community and Peer Influence
Financial Support	
Misconceptions and Education	Misunderstanding of Medical Procedures
Recommendations and Reflections	Importance of Early Diagnosis
	Positive Attitude and Coping Strategies
Time Factor	

LLETZ: Large Loop Excision of the Transformation Zone

### Theme 1: LLETZ intervention process and outcomes

Participants recounted their experiences leading up to their LLETZ biopsy results. They expressed these results differently; some referred to histology results as ‘not well’, indicating malignancy, ‘good’ or as ‘there is nothing that they (professionals) see’, implying benignancy. Some participants referred to it as cancer or the disease. Most participants experienced malignant outcomes, while others experienced benign outcomes.

*“It was taken to wherever, after it came back, I was called to say come and see what we have sent. It was found that it was not well (meaning malignant). In reality there is cancer out there”* (p.1).

*“After this sample was taken to be tested, I was told that I have cancer”* (p.2).

*“When they return, they say there is nothing they see in the womb”* (p.3).

Another participant, though cancer was diagnosed, expressed the prognosis experienced as:

*“Results came back, and I found that I got cancer of the cervix. I went to Mankweng [hospital] where I found that the cancer I have has just started and is not too long so that my womb can be removed”* (p.5).

One participant, when asked what the results were, replied:

*“Good (meaning benign)”* (p.7).

### Theme 2: Physical experiences

The physical experiences of the participants varied, with many reporting pain and discomfort associated with the procedure and recovery process. Participants described their experiences as follows:

“I *experienced some minor pains here (pointing at the lower abdomen). Sometimes I feel something in my tummy. I have been given some pain blocks*” (p.1).

“On *the day of treatment, I felt a little bit of pain the whole day*” (p.2).

One participant provided the aggravating factors behind the discomfort experienced and the length of time it took:

*“On the day when the sample is taken, you feel pain. Mhmm When you pass urine, you feel a bit of pain. Even when you bath. It took a week, but those muscles were pulling after two weeks. It was painful but unlike just after it was cut [sic]”* (p.7) *.*

Some participants felt no pain:

*“I accepted and did not have a problem. I am going to have a sample taken to hear what the problem is with me. It was done, and there was no pain* [turning the head sideways] *”* (p.4).

Despite the initial pain, many participants found that their symptoms improved post-procedure. As one noted:

“ *The pains that I felt post-procedure just disappeared*” (p.6).

Recovery was another critical sub-theme, with several participants discussing their healing process regarding pain, bleeding and tissue integrity. For instance:

*“The sample was taken; it was painful at that time, but it did not take long to heal”* (p.7).

*“I even asked if I would be admitted, and they said no, just go home. I was so shocked and still remember that when I left, I might have stayed for more than three hours outside thinking about this thing – it meant it would bring a lot of pain. It is better that I stay and feel it while I am still here until it came to me that as I was walking normally, not feeling pain, stepping well”* (p.6).

About bleeding, variance was also observed:

*“Blood came out, but not to stress me”* (p.1).

“ *That day I was cut. The following day, there was no blood*” (p.7).

For other participants, it took longer:

*“After I was done on Wednesday, Thursday, I was still bleeding. Thursday and Friday as well. Then I told myself that I would go on Monday if I bled on Sunday. On Sunday, I got up normally. Monday, it was finishing, and Tuesday was nothing. I did not go to the clinic”* (p.6).

One participant, aware of the impact HIV could have on tissue integrity, expressed concern and took proactive measures to ensure speedy recovery:

“… *as I did not understand how the wound will heal as it is inside…as a sore of an HIV person does not heal easily. I kept bathing with water and salt”* (p.7).

### Theme 3: Psychological Experiences

Participants reported a range of psychological responses to their diagnosis and treatment. Many displayed a notable level of acceptance and resilience, as illustrated by the following statements:

*“I accepted that what happened has happened. However, I am HIV positive as well. I indeed took a sample (referring to a pap smear), and the result came back, and I heard what was happening with my womb. This was not a problem”* (p.4).

*“It helped me as I was being admitted to the hospital monthly and was… I do not know if it is that I accepted; this helped me in spirit to heal. I no longer panic as the problem is being addressed. Since 6 ^th^ March, I came back today, I am no longer scared”* (p.6).

This acceptance often seemed linked to the support they received from their families and partners. However, anxiety and stress were also prevalent. One participant described her initial reaction to the diagnosis as follows:

*“I was stressed a little bit … hmm, whether it is the end of the world or will I be able to live again?” [sic]* (p.2).

Another participant, reacting to the introduction of surgical intervention, said:

*“Very scared. When I was told that today I am going to have a sample cut, not understanding how it is going to be done [sic]”* (p.3).

The uncertainty about the future and fear of a life-threatening illness were common themes:

*“I was stressed as I did not know whether this was cancer. As they delay, will I not die? I am bleeding; on the other hand, they are delaying. I was stressed. No peace. I thought I would die. Why are they not telling me what the problem is? When I come for results, they say there are none; I am not being checked. I was stressed. I got the results. I was right as I saw nothing serious. I know where I stand”* (p.3).

*“…the fear I had as…eee… I used to understand that if a sample taken inside… how will that be? how will I be? That means I will always be in pain? Not knowing what is happening*” (p.6).

*“I was scared as I did not understand how the wound will heal as it is inside”* (p.7).

The effect of certainty about health status was also expressed:

*“Yes, it helped a lot. Now I live knowing my situation”* (p.7).

### Theme 4: Psychosocial support

Family and partner support played a significant role in participants' experiences. One participant shared:

*“I was supported by my eldest daughter who said you should go to the hospital to know what disease you are suffering from and start with the treatment”* (p.2).

*“… but I had supporters who strengthened me. I withstood it until I got the results that I got cancer of the cervix, and they comforted me that I come to the hospital. They support me a lot, including others close to me so that I be right”* (p.5).

Other participants also emphasized the support received from their husbands:

*“My husband accompanied me and agreed that I have a sample taken”. “Wherever we go, we are together, and he knows all (*demonstrating with hands sign like brackets) *that I am”* (p.4).

*“I was not comfortable yes (nodding her head), but I accepted as he did accept and he supports me a lot”* (p.5).

Community and peer influence also emerged as an essential factor. Discussions with peers provided a source of comfort and shared experiences, as reflected by the following statement:

*“But you did not experience it. Yes, as we are sitting there (pointing to the side of the queue), we talk”* (p.3).

The participant who had a supportive husband learned about others' negative experiences while waiting in the queue:

*“Heee, my husband was always angry. Got a girlfriend, saying he cannot stay with me and bring another woman”* (p.3).

### Theme 5: Financial support

In addition to other necessary support, financial assistance for transportation to subsequent hospital visits for LLETZ procedures is crucial. Travel costs, including public transportation, can create a significant burden, making targeted financial help essential for ensuring patients can consistently attend their appointments and receive proper care.

“ *We also helped each other with money for transport. When one does not have, the other assists*” (p.4).

“ *Money… (rubbing thumb and pointing finger together) is what bothers a lot. If things are not complete, you got to come back again*” (p.5).

### Theme 6: Misconceptions and education

Participants demonstrated several misconceptions about the LLETZ procedure. These misconceptions were clarified through education. This highlights the importance of providing thorough pre-procedure counseling to alleviate unnecessary fears. For example, one participant initially feared the procedure, believing it to be more invasive than it actually was:

*“And some of the things that I did not know, I was thinking it means when they are done, I will be sutured and have a bandage on, but when they finish, they just told me they are finished”* (p.6).

Another misunderstanding was about the intervention site, affecting intervention outcome expectations. Participant attributes three miscarriages to HIV and vaginal bleeding. The participant hoped that the non-LLETZ-related bleeding would be addressed, mistakenly believing the procedure was done in the uterus: This is clear from the third participant’s experience:

*"When they returned, they said there was nothing visible in the womb [sic]. The bleeding should have stopped after they removed the sore, so we did not understand why it continued. This indicates that something is preventing my children from staying in the womb [sic], and I would like to learn more about this bleeding, as it is causing me issues"* (p.3).

### Theme 7: Recommendations and reflections

Participants provided several recommendations based on their experiences, emphasizing the importance of early diagnosis and maintaining a positive attitude:

*“If I do a pap smear and they see what the problem is when it is still early (emphasizing using hands). If the sore was cancerous, it means I would have left it to be worse and incurable”* (p.3).

Another participant encouraged others to take proactive health measures:

*“And other women test (saying it strongly and nodding her head while standing up at the same time.) go and do a pap smear to get womb results and know what is going on”* (p.5).

Maintaining a positive attitude was also crucial, as one participant advised:

*“Let us accept it as it is. It is not the end of the world. We will continue to live and go ahead”* (p.4).

*“I would say if you got a problem, share it with someone. If you find it is not easy, speak to your children. Your children will not be like outside people. They will understand. They will support you”* (p.7).

This perspective was often linked to the support they received and their coping strategies.

Acceptance of LLETZ diagnostic outcomes and disclosure are identified as coping strategies:

*“I can explain that women, even if we come across this situation (pointing within* the space), *whatever they say- we got cancer or what? Let us accept it as it is. It is not the end of the world. We will continue to live and go ahead”* (p.4).

*“… at home, they know I was not well. I told them that I accepted the situation and I would come out of this, and I did it. They were ready to join and support me.”* (p.6).

### Theme 8: Time factor

Alongside their experiences, women also highlighted the significance of time. LLETZ biopsy histology results showed malignancy for one participant, who took decisive action that led to positive outcomes:

*“…disease was detected when it was still early.* I *took a step for treatment, went to Mankweng (Provincial Hospital), to Polokwane (Provincial Hospital) and was given treatment until I became a good and better person*” (p.2).

Subsequent participants agree that prompt action leads to positive outcomes:

*“Let us get used to going to the clinic and not leave things to be worse”* (p.3).

*“If I do a pap smear and they see what the problem is [sic] when it is still early (emphasizing using hands). If the sore was cancerous, it means I would have left it to be worse and incurable. …going to the clinic assisted in being diagnosed while in early stages and was assisted”* (p.3).

Once again, the time factor was highlighted concerning the turnaround time for results:

*“I was given a date for results and found that it takes long, (showing sadness facially and voice going low”* (p.3).

“ *I found like it takes time. When I come, they say there is none when I come, they say there is none until I get them – the results. I think is last month (referring to March 2024)*” (p.3).

The findings of the thematic analysis underscore the intricate and diverse experiences of women living with HIV and undergoing LLETZ treatment. Essential themes from the participants’ narratives encompass psychological resilience, physical discomfort and the significant role of social support. Misconceptions about the procedure highlight the necessity for comprehensive patient education. The recommendations put forward by the participants emphasize the importance of early detection and a positive outlook as essential components of managing their health and well-being. These insights can provide valuable guidance to healthcare professionals and policymakers in enhancing support systems and educational resources for women facing similar treatments.

## Discussion

The study’s findings provide a comprehensive view of the multifaceted impact of LLETZ on women with HIV, capturing their physical, psychological, and social experiences. The primary goal of LLETZ is to excise premalignant lesions while allowing histological examination to determine malignancy status. This approach is particularly critical for women living with HIV, who face an elevated risk of rapid lesion progression compared to HIV-negative counterparts ( Van Bogaert, 2014; Bambury et al., 2013). However, delays between pap smear results and histology confirmation often exacerbate psychological distress in this vulnerable group, highlighting the need for faster diagnostic timelines and improved emotional support to minimize the strain during the waiting period ( Yap et al., 2020). These delays add a layer of complexity to an already challenging physical experience for patients undergoing LLETZ.

Physical discomfort was widely reported among participants, emphasizing the importance of personalized care approaches. Pain levels varied significantly, which underscores the subjective nature of post-procedural recovery. Tailored care, including individualized pain management strategies and patient education on self-care, has positively influenced recovery and patient satisfaction ( Goldstein et al., 2024). These physical challenges often intertwine with psychological experiences, adding further complexity to recovery and highlighting the need for integrated support.

Psychologically, the procedure profoundly impacted participants, with many experiencing anxiety and stress, particularly regarding their diagnosis and potential prognosis. While some participants displayed resilience, the emotional burden of awaiting uncertain outcomes suggests a need for enhanced counseling ( Sparić et al., 2019). Studies have shown that psychosexual health is a critical area affected by cervical excisional treatments, impacting long-term emotional recovery. Such counseling could prepare patients for potential emotional challenges and equip them with coping mechanisms, addressing the dual demands of physical and psychological recovery. This integration of mental health support into physical healthcare is vital, especially for HIV-positive patients facing a challenging prognosis (Debeudrap et al., 2019). Alongside counselling, social support from family and community plays a crucial role in aiding recovery.

Psychosocial support, especially from family and partners, was a significant factor in managing the treatment process for participants. This support reduced feelings of isolation and encouraged adherence to treatment recommendations. Partner engagement, in particular, reinforces the importance of a collaborative healthcare approach and underscores the need for inclusive counselling strategies that can positively affect reproductive health outcomes ( Finocchario-Kessler et al., 2016). Community and peer support also played a role, indicating the potential of shared experiences to foster a stronger recovery network. These social supports, however, must be complemented by practical resources to address financial barriers.

Financial challenges were a persistent obstacle, with participants highlighting transport costs and other treatment expenses. Financial instability often restricts access to continuous care, underscoring the need for systemic support for low-income patients undergoing LLETZ. This finding aligns with studies that emphasize the critical role of financial stability in ensuring treatment adherence, particularly within underserved communities ( Van der Heijden et al., 2015; Owenga & Nyambedha, 2018). HPV testing has also been shown to play a valuable role in follow-up strategies post-LLETZ for cervical intraepithelial neoplasia (CIN) ( Van der Heijden et al., 2015). Economic support and social backing can help mitigate the material and emotional burdens patients face, but this must be underpinned by effective communication from healthcare providers.

Misunderstandings about the procedure were common, underscoring the necessity of pre-procedure education. Many patients had misconceptions about the invasiveness and expected outcomes of LLETZ, which, if unaddressed, may lead to unnecessary fear or anxiety. Thorough education on the procedure, aided by anatomical diagrams, can clarify the procedure’s intent and expected effects, reducing patient apprehension ( Manga et al., 2024). Education is especially relevant in preparing patients for proactive health behaviors, which participants identified as a key recommendation.

Participants emphasized the importance of early detection and regular screenings, echoing policy initiatives that advocate for increased access to cervical cancer screening, particularly for high-risk populations like HIV-positive women. Early diagnosis improves outcomes and aligns with healthcare systems' growing emphasis on preventive care. Routine screening facilitates the timely identification of cervical abnormalities, preventing progression and enabling less invasive interventions ( Goldstein et al., 2024). However, preventive care must be supported by effective follow-up processes, particularly concerning the timing of result delivery.

The timing of result delivery emerged as a critical area of concern, with prolonged wait times for histology results contributing to heightened patient anxiety. Short-term diagnostic timelines could alleviate psychological distress and foster a more predictable care experience. A standardized turnaround time may also improve patient satisfaction, addressing a crucial gap in service delivery for high-risk and resource-limited populations ( Manga et al., 2024). This underscores the need for a holistic approach to care, integrating timely, supportive, and comprehensive measures.

This study emphasizes the importance of a holistic approach to the LLETZ procedure for women living with HIV, addressing medical needs as well as emotional, social, and economic aspects of care. Integrating patient counseling, financial assistance, comprehensive education, and timely diagnostics into the LLETZ care pathway could significantly improve treatment outcomes, reduce procedural stress, and enhance the overall healthcare experience. The research examines the implications of the LLETZ procedure on women's health and quality of life, offering recommendations to enhance well-being and potentially increase life expectancy.

To achieve these goals, the study underscores the necessity of open communication within families, encouraging patients to discuss their health conditions with their children for better support. Comprehensive pre-procedure counseling is deemed essential for minimizing patient uncertainty. It also calls for investigating the waiting period for histological examination following LLETZ at Vhembe District Hospital to identify factors impacting turnaround times. Involving partners in the healthcare journey and utilizing visual aids, such as diagrams of the female reproductive system, can clarify the procedure and foster understanding. Lastly, the importance of continuous education for healthcare professionals is highlighted to ensure that future workers are equipped to support women undergoing LLETZ. Overall, these recommendations aim to improve healthcare experiences and outcomes for women through collaboration among families, health professionals, and educational institutions.

## Conclusions

This study emphasizes the critical need for comprehensive education and effective communication for women living with HIV who undergo LLETZ procedures, as significant gaps in understanding can negatively impact patient experiences and expectations. By identifying themes such as misconceptions and the psychological effects of uncertainty, the findings highlight the necessity for targeted interventions that address the unique psychosocial challenges faced by this population, particularly in reproductive health. Moving forward, healthcare providers, researchers, and educators must prioritize developing tailored educational programs and strategies that foster continuous patient engagement, clarify misconceptions and establish realistic expectations. Ultimately, the recommendations from this study provide a foundation for future research and practice aimed at minimizing uncertainty and improving the quality of care for women navigating the complexities of living with HIV and undergoing such medical interventions, thus advancing equitable health outcomes in the years to come.

## Ethical considerations & consent

The study received ethical approval and clearance from the University of Venda Human and Clinical Trial Research Ethics Committee, which issued the Ethical Clearance Certificate (Ethical Clearance Number: FHS/23/PH/56/1801) approved on 18 January 2024. Eligible participants received information about the study's purpose, methods, and their role in it, along with a consent form in their native languages. After agreeing, written consent was obtained.

## Data Availability

Due to ethical considerations and to protect the confidentiality of participants, the data collected in this study is not publicly available. The dataset contains sensitive personal information related to women living with HIV who have undergone LLETZ and sharing identifiable or raw data could compromise participant privacy. Access to the data may be considered upon reasonable request, subject to ethical and institutional approval. Researchers wishing to access the data must submit a formal request to the University of Venda Human and Clinical Trial Research Ethics Committee, outlining the intended use, data protection measures, and compliance with confidentiality protocols. Requests will also require approval from the Limpopo Department of Health and the management of the participating hospitals. Any granted access will be provided under strict conditions to ensure participant anonymity and data security, including restricted viewing in a secure environment or de-identified summaries of findings. For further inquiries, interested parties can contact the University of Venda Human and Clinical Trial Research Ethics Committee or the corresponding author of this study. OSF: Experiences of women living with HIV who underwent long loop excision of the transformation zone (LLETZ) in selected hospitals of Vhembe district, Limpopo province, South Africa. https://doi.org/10.17605/OSF.IO/BKNS5 ( Makhado and Sikhwetha 2025) The project contains the following extended data:
1. Supplement 1 interview guide Data are available under CC0 1.0 Universal license
