# Supplementary material for: Experiences of women living with HIV who underwent long loop excision of the transformation zone (LLETZ) in selected hospitals of Vhembe district, Limpopo province, South Africa
Source: F1000Res. 2025 Sep 5;14:382. Originally published 2025 Apr 2. [Version 2] doi: 10.12688/f1000research.158796.2 (PMC12489456; doi:10.12688/f1000research.158796.2)
Supplement: Peer review history

# Reviewer report: Reviewer response for version 2

**DOI:** 10.5256/f1000research.187671.r414049

**Published:** 2025-10-01

**Authors:** Tintswalo Victoria Nesengani

**Affiliations:** 1 University of Pretoria, Pretoria, Gauteng, South Africa

The Abstract is clearly structured and covers aspects such as the Background information, Methods, Results and Conclusions, giving a clear link between the different aspects, as well as an indication of what the Article talks about.

 The Introduction clearly introduces the topic, and relevant, current literature had been used. Different views in relation to the topic were given as discussed by various authors. Statistics from different countries globally pertaining to the topic were highlighted to indicate the magnitude of the problem of cervical cancer; which also shows a clear understanding of such a problem by the researcher, including it's impact to women of reproductive age in various communities.

 Methods used were suitable for the study. Data analysis was coherent to the research design, with a clear indication of how the themes were derived.

 The results are well presented, with participants quotations supporting the derived themes.

 The discussion is clearly written, with literature control well applied. Current, relevant sources have been used. Conclusions are well written and summarizes the themes as well as the recommendations of the study.

 Ethical considerations are well applied.

 Acknowledgements and References are well written.

 The Article complies to Scientific writing and the Author Guidelines provided.

Is the work clearly and accurately presented and does it cite the current literature?

Yes

If applicable, is the statistical analysis and its interpretation appropriate?

Yes

Are all the source data underlying the results available to ensure full reproducibility?

Yes

Is the study design appropriate and is the work technically sound?

Yes

Are the conclusions drawn adequately supported by the results?

Yes

Are sufficient details of methods and analysis provided to allow replication by others?

Yes

Reviewer Expertise:

Human caring

I confirm that I have read this submission and believe that I have an appropriate level of expertise to confirm that it is of an acceptable scientific standard.

# Reviewer report: Reviewer response for version 1

**DOI:** 10.5256/f1000research.174437.r375659

**Published:** 2025-06-12

**Authors:** Chemtai Mungo

**Affiliations:** 1 University of North Carolina, Chapel Hill, Carolina, USA

**Introduction**

 Please restructure the Introduction to follow the standard “funnel” approach—beginning with a broad overview of the field, narrowing to the specific knowledge gap, and ending with a clear statement of the study objective. At present the objective is placed in the opening paragraph, which disrupts the logical flow and makes the section difficult to follow. See this reference on how to structure a scientific paper: (Mensh B & Kording K, 2017)- **Ref 1.**

- Please relocate the statement of the study objective to the final paragraph of the Introduction, in keeping with conventional manuscript structure.
- The link provided for “Jo’s Cervical Cancer Trust (2020)” is inactive and the source does not appear to be a peer-reviewed reference. Please replace it with a scholarly citation that reports LLETZ efficacy, and specify whether the quoted success rate applies to HIV-negative or HIV-positive women. Published data indicate substantially lower cure rates in HIV-positive cohorts.
- Paragraph 3 of the Introduction contains a typographical error: “78,6 %” should read “78.6 %”.
- In the same paragraph, the phrase “the absence of specific recommendations for this high-risk group” is unclear, given your earlier statement that LLETZ is recommended for CIN 2/3 in HIV-positive women. Please clarify which recommendations are lacking.

**Methods**

- Pre-test section: Omit routine details such as checking pens, notebooks, or recorder batteries; these are assumed and do not add value to the manuscript.

**Results**

- Do not capitalise “participant seven” or similar descriptors within sentences; they are common nouns.
- Under Theme 1, the term “benignancy” is uncommon in cervical cancer literature. Consider defining it or substituting a more widely used term.

 

 This study offers valuable insights into women’s experiences with LEEP/LLETZ in high-burden regions—an issue central to achieving the WHO 90-70-90 cervical cancer elimination targets. While the results are noteworthy, the Introduction and Methods sections require substantial revision to meet publication standards. Please address the specific comments above and revise the manuscript, accordingly, ensuring that refinements in these sections are reflected throughout the paper.

Is the work clearly and accurately presented and does it cite the current literature?

No

If applicable, is the statistical analysis and its interpretation appropriate?

Not applicable

Are all the source data underlying the results available to ensure full reproducibility?

Yes

Is the study design appropriate and is the work technically sound?

Yes

Are the conclusions drawn adequately supported by the results?

Yes

Are sufficient details of methods and analysis provided to allow replication by others?

Yes

Reviewer Expertise:

Secondary prevention of cervical cancer in LMICs

I confirm that I have read this submission and believe that I have an appropriate level of expertise to state that I do not consider it to be of an acceptable scientific standard, for reasons outlined above.

## References

[1] : Correction: Ten simple rules for structuring papers. *PLOS Computational Biology* .2017;13(11) : 10.1371/journal.pcbi.1005830 10.1371/journal.pcbi.1005830 PMC567951229121064

# Response

**Published:** 2025-09-02

**Authors:** LUFUNO MAKHADO

**Affiliations:** Public Health, University of Venda, Thohoyandou, Limpopo, South Africa

**Point to Point Response to Reviewers**

 We sincerely thank the reviewers for their thoughtful and constructive comments, which have helped us improve the clarity, coherence, and scholarly quality of our manuscript. Below, we respond point by point, indicating the revisions made.

**Reviewer Comment 1**

 “Please restructure the Introduction to follow the standard ‘funnel’ approach—beginning with a broad overview of the field, narrowing to the specific knowledge gap, and ending with a clear statement of the study objective. At present, the objective is placed in the opening paragraph, which disrupts the logical flow and makes the section difficult to follow. See Mensh & Kording, 2017.”

**Response**

 We thank the reviewer for this important suggestion. We have restructured the Introduction to follow the funnel approach. It now begins with the global burden of cervical cancer, narrows to the specific issue of LLETZ treatment in HIV-positive women, highlights the lack of tailored recommendations and the absence of qualitative data on women’s lived experiences, and concludes with a clear statement of the study objective in the final paragraph. This aligns with the guidance of Mensh & Kording (2017).

**Reviewer Comment 2**

 “Please relocate the statement of the study objective to the final paragraph of the Introduction, in keeping with conventional manuscript structure.”

**Response**

 We agree with this recommendation. The statement of the study objective has been moved from the opening paragraph to the concluding paragraph of the Introduction to ensure a conventional scientific structure and logical flow.

**Reviewer Comment 3**

 “The link provided for ‘Jo’s Cervical Cancer Trust (2020)’ is inactive, and the source does not appear to be a peer-reviewed reference. Please replace it with a scholarly citation that reports LLETZ efficacy, and specify whether the quoted success rate applies to HIV-negative or HIV-positive women. Published data indicate substantially lower cure rates in HIV-positive cohorts.”

**Response**

 We appreciate this observation. The non-peer-reviewed source has been replaced with a scholarly citation: Debeaudrap et al. (2019), which reports pooled cure rates of ~78.6% in HIV-positive women compared to >90% in HIV-negative women. The text has been revised to clearly specify these differences.

**Reviewer Comment 4**

 “Paragraph 3 of the Introduction contains a typographical error: ‘78,6 %’ should read ‘78.6 %’.”

**Response**

 We thank the reviewer for identifying this typographical error. It has been corrected to “78.6 %.”

**Reviewer Comment 5**

 “In the same paragraph, the phrase ‘the absence of specific recommendations for this high-risk group’ is unclear, given your earlier statement that LLETZ is recommended for CIN 2/3 in HIV-positive women. Please clarify which recommendations are lacking.”

**Response**

 We appreciate this request for clarification. The text has been revised to specify that while LLETZ is recommended for CIN2/3 in HIV-positive women, there is an absence of tailored recommendations for follow-up intervals, recurrence management, and long-term monitoring in this group.

**Reviewer Comment 6**

 “Pre-test section: Omit routine details such as checking pens, notebooks, or recorder batteries; these are assumed and do not add value to the manuscript.”

**Response**

 We agree with this suggestion. The Pre-test section has been streamlined, and routine details about equipment checks have been removed. The section now focuses only on methodological relevance, such as the refinement of probing techniques.

**Reviewer Comment 7**

 “Do not capitalise ‘participant seven’ or similar descriptors within sentences; they are common nouns.”

**Response**

 We thank the reviewer for pointing this out. All instances of “Participant [number]” have been changed to lowercase (e.g., “participant seven”) throughout the manuscript.

**Reviewer Comment 8**

 “Under Theme 1, the term ‘benignancy’ is uncommon in cervical cancer literature. Consider defining it or substituting a more widely used term.”

**Response**

 We appreciate this observation. The term “benignancy” has been replaced with the more widely accepted phrase “benign outcome” to ensure clarity and alignment with standard terminology in cervical cancer literature.

**Conclusion**

 We are grateful to the reviewers for their careful reading and constructive feedback. The suggested revisions have improved the manuscript’s structure, clarity, and alignment with scholarly standards. We hope that the revised version satisfactorily addresses all concerns.

 Kindest regards

**Lufuno.**

# Reviewer report: Reviewer response for version 1

**DOI:** 10.5256/f1000research.174437.r375660

**Published:** 2025-04-12

**Authors:** Mygirl Lowane

**Affiliations:** 1 Sefako Makgatho Health Sciences University, Ga-Rankuwa, South Africa

Thank for the opportunity to peer review this manuscript titled: Experiences of women living with HIV who underwent long loop excision of the transformation zone (LLETZ) in selected hospitals of Vhembe district, Limpopo province, South Africa.

 I must commend that it is a well written manuscript in line with the journal scope. The abstract gave a satisfactory overview of all aspects and summarizes the entire paper.

 Page 3: paragraph 1, LLETZ – should be written in full before the abbreviation. I suggest that paragraph 1&2 to be merged as it addressing the same content. Furthermore, to my suggestion, the symbol “%” which is outside the brackets should be written in full word as “per cent”.

 From the background to discussion, the authors made a good attempt at providing a contextual platform, building the interest of the reader on paucity of integration of the need to explore Experiences of women living with HIV who underwent long loop excision of the transformation zone (LLETZ) in selected hospitals of Vhembe district, Limpopo province, South Africa.

 The literature used in this paper gives the evidence of sufficient engagement with the relevant literature. The authors demonstrate adequate interpretation of relevant literature with depth in the arguments.

 The methodology: The use of qualitative method and phenomenological design by the authors is appropriate and this section is well done. The method, collection, and analysis of data is detailed enough to allow others to replicate them. The themes and the participants' quotes complement each other.

 Discussions also focused on the results of the study, including its implications and what the study adds to the knowledge.

 In summary, I found that objectives and research methods interact, that the results are clearly presented, that discussions are supported by literature, and that the conclusions justify the content of this document.

 I therefore Approved with very minor changes required.

Is the work clearly and accurately presented and does it cite the current literature?

Yes

If applicable, is the statistical analysis and its interpretation appropriate?

Not applicable

Are all the source data underlying the results available to ensure full reproducibility?

Yes

Is the study design appropriate and is the work technically sound?

Yes

Are the conclusions drawn adequately supported by the results?

Yes

Are sufficient details of methods and analysis provided to allow replication by others?

Yes

Reviewer Expertise:

Epidemiological studies,  Mental Health, Mother and child, HIV, STI, TB Sexual and reproductive Health Immigrant studies

I confirm that I have read this submission and believe that I have an appropriate level of expertise to confirm that it is of an acceptable scientific standard.
